# Supplementary material for: Task-induced topological and geometrical changes in whole-brain dynamics predict cognitive individual differences
Source: bioRxiv. 2026 Apr 22:2026.04.19.719533. Preprint. [Version 1] doi: 10.64898/2026.04.19.719533 (PMC13131634; doi:10.64898/2026.04.19.719533)
Supplement: 1 [file NIHPP2026.04.19.719533V1-supplement-1.pdf]

bioRxiv preprint doi: https://doi.org/10.64898/2026.04.19.719533; this version posted April 22, 2026. The copyright holder for this preprint (which was not certified by peer review) is the author/funder, who has granted bioRxiv a license to display the preprint in perpetuity. It is made available under aCC-BY 4.0 International license.

Supplementary Figures

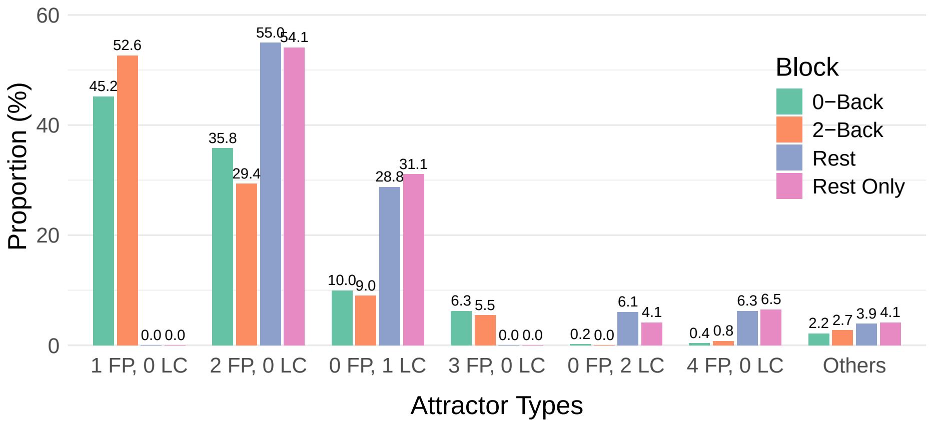

Figure S1: **Taxonomy of dynamics across resting-state only models and joint models in each condition.** The resting-state only models were used in the analysis in Section 3.5.

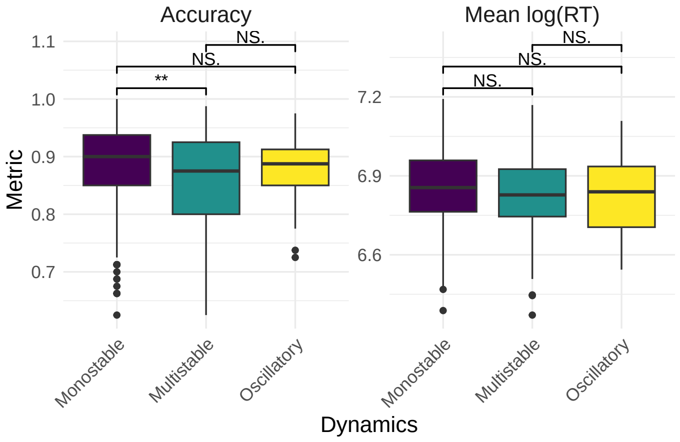

Figure S2: **Behavioral differences across participants with different dynamics in 2-back condition.** ANOVA was significant for accuracy ($F(2, 508) = 5.507$, $p = 0.00431$) and marginally significant for log reaction time ($F(2, 508) = 2.601$, $p = 0.0752$). Only the differences between monostable and multistable groups for accuracy was significant (Tukey's HSD, estimate = 0.0237, CI: $[0.0067, 0.0407]$, adjusted $p = 0.0032$). "N.S.": not significant.

bioRxiv preprint doi: https://doi.org/10.64898/2026.04.19.719533; this version posted April 22, 2026. The copyright holder for this preprint (which was not certified by peer review) is the author/funder, who has granted bioRxiv a license to display the preprint in perpetuity. It is made available under aCC-BY 4.0 International license.

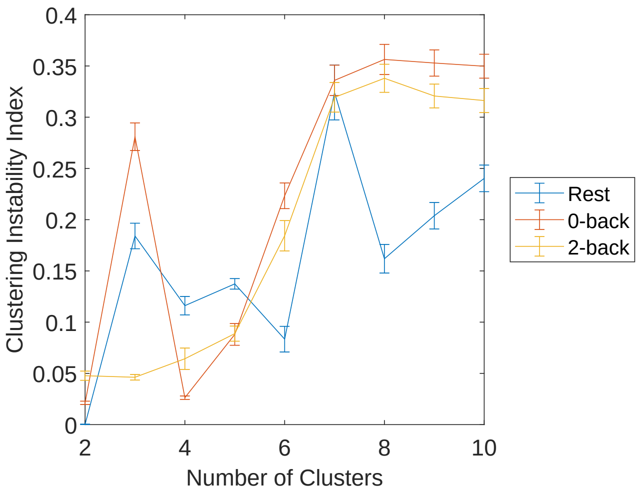

Figure S3: **Clustering instability for attractors in each condition.**

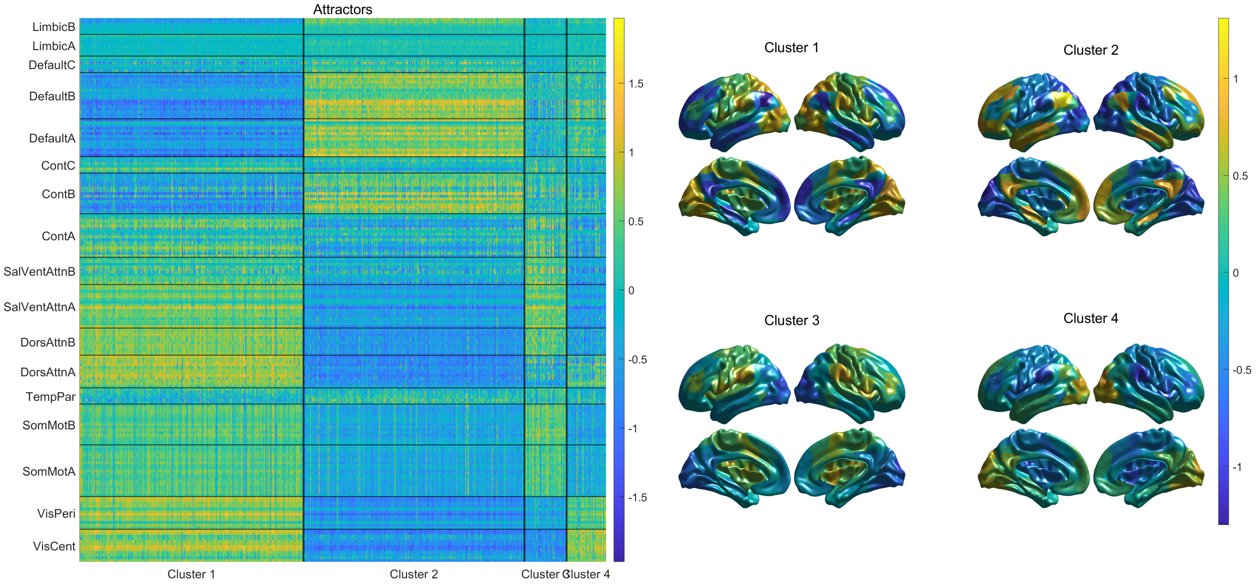

Figure S4: **Clustering resting state attractors.** Left: all resting state attractors. Each row represents one region and each column represents one attractor. Color indicates the coefficient (activation). Right: cluster centroids visualized as activation pattern over the cortex.

bioRxiv preprint doi: https://doi.org/10.64898/2026.04.19.719533; this version posted April 22, 2026. The copyright holder for this preprint (which was not certified by peer review) is the author/funder, who has granted bioRxiv a license to display the preprint in perpetuity. It is made available under aCC-BY 4.0 International license.

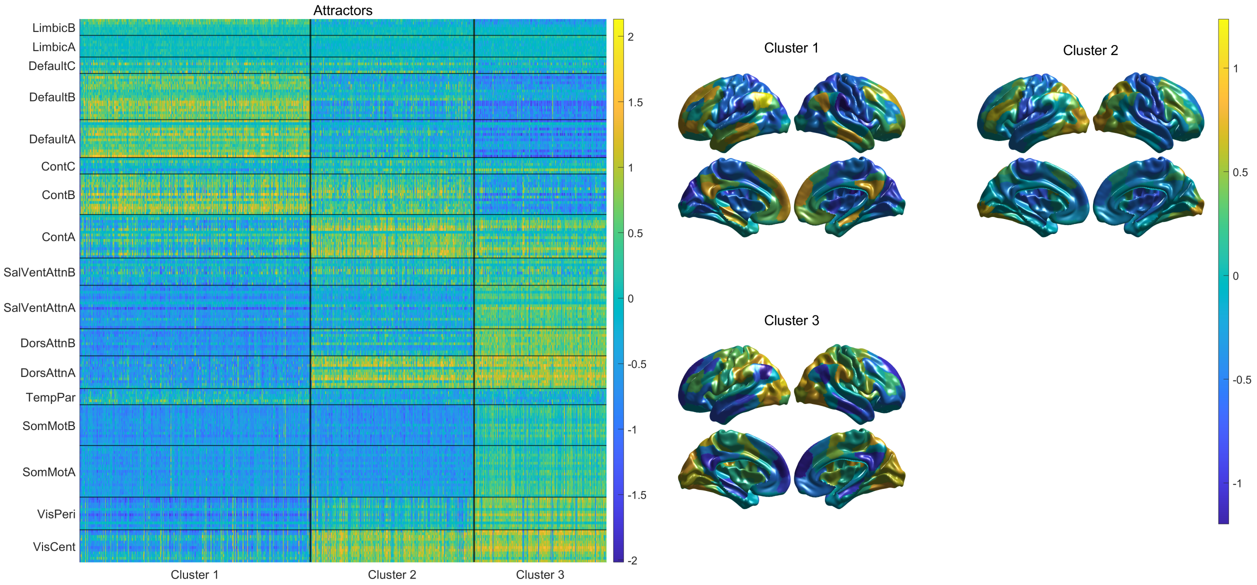

Figure S5: **Clustering 2-back condition attractors.**
